# Should ICSI be implemented on patients with poor-quality embryos in the previous IVF cycle?

**DOI:** 10.1016/j.heliyon.2023.e17996

**Published:** 2023-07-07

**Authors:** Jiang Wang, Shun Xiong, Yang Gao, Fei Xia, Biao Wei, Jiayi Zou, Guoning Huang, Wei Han

**Affiliations:** a Chongqing Key Laboratory of Human Embryo Engineering, Center for Reproductive Medicine, Women and Children's Hospital of Chongqing Medical University, Chongqing, China; b Chongqing Clinical Research Center for Reproductive Medicine, Chongqing Health Center for Women and Children, Chongqing, China

**Keywords:** Poor-quality embryo, Utilization rate, Clinical outcome, IVF, ICSI

## Abstract

This study was to evaluate whether Intracytoplasmic sperm injection (ICSI) can improve the quality of embryo in patients with poor-quality embryos in the previous In-vitro fertilization (IVF) cycle, which was cancelled before transfer. This was a retrospective cohort study of 178 IVF and 158 ICSI cycles for patients with poor-quality embryos in the previous IVF cycle in the Center for Reproductive Medicine, Women and Children's Hospital of Chongqing Medical University from March 2016 to June 2022. The 2 PN rate, oocyte utilization rate , high-quality embryo rate and clinical pregnancy rate were compared between the two groups. Furthermore, the implantation rate, miscarriage rate and cycle cancelation rate were measured and compared. ICSI resulted in a comparable 2 PN rate, oocyte utilization rate and cycle cancelation rate with IVF. The high-quality embryo rate of ICSI group was significantly higher than that of IVF group (5.56% vs. 2.60%, P < 0.05). Eventually, a total of 239 patients performed embryo transfer. ICSI resulted in a significantly higher clinical pregnancy rate (55.56% vs. 40.98%, P < 0.05) compared with IVF, however, there were no notable differences in miscarriage rate and implantation rate. The present study suggested that ICSI significantly improved the high-quality embryo rate and clinical pregnancy of the patients with poor-quality embryos in the previous IVF cycle. Prospective randomized controlled trials are needed to further verify.

## 1 Introduction

Intracytoplasmic sperm injection (ICSI) involves the microinjection of spermatozoa into mature oocytes. It was first introduced in 1992 to treat infertility in couples with severe male-factor [1]. Despite its early introduction, ICSI was developed for male factor infertility more recently and introduced as a regular choice of assisted reproductive technology (ART). European in-vitro fertilization (IVF) monitoring report that ICSI remains the most used. In the 1 007 598 ART cycle, including 400 375 with ICSI in 2018 [2]. The expanded indications for use of ICSI include low oocyte yield, advanced maternal age, prior fertilization failure or poor fertilization with conventional insemination, poor-quality oocytes, PGT, and so on [3]. However, controversy exists whether it is beneficial to use ICSI for the indications mentioned above. Some studies showed no significantly improved clinical outcomes in these couples undergoing ICSI [4,5]. In the meantime, other studies demonstrated that embryo quality and clinical outcomes in couples suffered from non-male factors were improved by ICSI compared with conventional IVF [6,7]. These inconsistent results may be due to the different study design, sample sizes, statistical methods, and the difference in patients involved.

This study focused on a subpopulation of patients with none-male factor infertility and failure in their previous IVF cycle owing to poor-quality of embryo. Failure in the previous IVF cycle for poor-quality embryos implied that these patients had a possibility of harvesting poor-quality embryo in the incoming IVF cycle. The intrinsic factor of oocytes was the predominant cause. So far, there is no effective way to improve embryo quality. Peng et al. demonstrated that ICSI resulted a significant high-quality embryo rate [8], and the study by Kim et al. showed that ICSI improved the embryo quality in patients with non-male factor infertility [9]. Therefore, ICSI may be preferred for the subpopulation of patients with poor-quality embryos. However, evidence on the effectiveness of ICSI in this subpopulation of patients is currently lacking. The present study aimed to evaluate the effect of the insemination method on the embryo quality in non-male infertile patients with poor-quality embryos in the previous IVF cycle.

## 2 Materials and methods

### 2.1 Patients’ eligibility criteria

In the retrospective cohort study, data were routinely collected from the Center for Reproductive Medicine, Women and Children's Hospital of Chongqing Medical University from March 2016 to June 2022. The study was approved by the Ethics Committee of Women and Children's Hospital of Chongqing Medical University (number: 2020-RGI-09). The fertilization method was decided by the doctors and patients in agreement based on the previous infertility history. Inclusion criteria were patients who had poor-quality embryos (embryos would be scored D and do not recommend to transfer) and non-male factor infertility. Exclusion criteria included: (1) less than five oocytes retrieved, (2) 2 PN rate less than 60%, (3) egg donor, (4) patients with preimplantation genetic diagnosis. A total of 336 couples were included in this study. ICSI was performed on 158 couples, and IVF was performed on 178 couples. This study was approved by the institutional review boards of Women and Children's Hospital of Chongqing Medical University.

### 2.2 Ovarian hyperstimulation and oocyte retrieval

Patients underwent standard controlled ovarian hyperstimulation (COH) protocol. Depending on their ovarian reserve, antagonist protocol, gonadotrophin-releasing hormone agonist (GnRHa), short and long down-regulation, or microdose flare protocol was selected and applied. Serum estradiol levels (E2) were monitored and the oocyte growth was evaluated with transvaginal ultrasound. When a minimum of 2 follicles reached 18 mm in mean diameter 1 × 10^4^ units of human chorionic gonadotropin (hCG) was injected to trigger oocyte maturation. Oocyte retrieval was carried out approximately 36–37 h after the hCG injection by a transvaginal ultrasound-guided puncture of the follicles.

### 2.3 Semen preparation and in vitro fertilization

After abstinence for 3–7 days, an ejaculate sample was obtained on the day of retrieval. When the semen liquefied, the sperm quality was assessed according to the World Health Organization (WHO) 2010 guidelines [10]. The density gradient centrifugation was used to prepare the sperm.

Conventional IVF was performed using the microdrop method. Approximately 3–4 h post-retrieval, 1–2 oocytes were added in the microdrop with 1.0 × 10^5^ motile sperm and co-incubated overnight. For oocytes inseminated with ICSI, cumulus cells were denuded 2 h after oocyte retrieval via hyaluronidase treatment. The ICSI was used to inseminate the Metaphase II oocytes (MII) with the spouse's sperm at least 1 h after removing the cumulus cells. After evaluated under a microscope at 400× magnification, high morphologic qualities and dynamic features sperm selected for ICSI, following the World Health Organization (WHO) 2010 guidelines [10]. Then zygotes were cultured in pre-equilibrated cleavage medium G1 (Vitrolife, Sweden).

### 2.4 Embryo evaluation and transfer

Around 12–18 h after insemination, the oocytes were examined for successful fertilization. The fertilization rate was defined as the number of oocytes with 2 PN divided by the number of cumulus-oocyte complexes. Embryonic development was evaluated on day 2 and 3, respectively. Classification of embryo quality was based on the Istanbul consensus [11]. High-quality embryos (embryos would be scored A) were 2 PN (2 pronucleus) origin, 4 cells on day 2, more than 6 cells on day 3, equally sized blastomeres and ≤10% fragmentation and no multinucleation; The available embryos (embryos would be scored B and C) were 2 PN origin, more than 6 cells and fragmentation <30%; Poor-quality embryos (embryos would be scored D and do not recommend to transfer) consist of all the rest. One or two available embryos on day 3 were transferred based on the patient condition, and high scored embryos were preferred choice to transplantation. Pregnancy was assessed using serum hCG assay 14 days after the embryos transfer. Additionally, clinical pregnancy was defined as the presence of a gestational sac with fetal heartbeat screening 28 d after the embryos were transferred.

### 2.5 Statistical analysis

Statistical analysis was performed using SPSS Statistics (version 22.0). The normality of distribution of continuous variables was tested by kurtosis and skewness test. Continuous variables were presented as mean ± standard deviation (SD) and were by Student's t-test. Analysis involving percentages and proportion for categorical variables was done using a Chi-square test. P < 0.05 was considered statistically significant.

## 3 Results

### 3.1 Patients baseline characteristics

The baseline characteristics of the ICSI and IVF groups were similar, except BMI of the female patients (p < 0.05). BMI was higher in the ICSI group than those in the IVF group, which is a negative factor for the formation of high-quality embryos in ICSI cycle and ART clinical outcome [12]. The constituent ratio of the infertility reasons in patients were not significantly different (p > 0.05). In addition, all patients underwent standard ovarian stimulation protocol, and there was also no difference in the constituent ratios (p > 0.05) (Table 1).

**Table 1:** Basal characteristics of the patients and ovarian stimulation.

	ICSI	IVF	ICSI vs. IVF
	n = 158 (47%)	n = 178 (53%)	p-Value
Female age (years)	32.45 ± 4.30	32.22 ± 4.09	0.615
Duration of infertility (years)	5.54 ± 3.57	5.64 ± 3.67	0.808
AMH (ng/ml)	3.06 ± 2.09	3.14 ± 2.64	0.774
BMI	22.11 ± 2.44	21.42 ± 2.68	0.014*
Basal serum FSH (IU/ml)	5.75 ± 1.75	5.70 ± 1.90	0.819
Infertility reason			0.448
Tubal factor	137	151	
Endometriosis	13	11	
Unexplained infertility	3	4	
Other reason	5	12	
Ovarian stimulation protocol			0.931
Antagonist protocol	46.83%	50.56%	
Long protocol	10.13%	8.43%	
Short protocol	26.58%	24.72%	
Super long-term protocol	10.76%	9.55%	
Other protocol	5.70%	6.74%	
Daily dose of gonadotropins	214.48 ± 58.09	224.65 ± 60.66	0.119
Length of stimulation	9.49 ± 2.15	9.50 ± 2.21	0.979
Gonadotropin units (IU)	2115.38 ± 747.22	2223.19 ± 821.02	0.211
Male factor			
Male age (years)	34.23 ± 5.06	34.45 ± 4.52	0.681
Normal sperm morphology (%)	4.75 ± 1.35	4.94 ± 1.37	0.187

Two-sample independent *t*-test or person χ2 test.

* Significant difference between IVF and ICSI groups, P < 0.05.

### 3.2 Embryonic outcome and clinical outcome

There was no statistically significant difference in the number of oocytes retrieved and absolute number of mature (MII) oocytes. The 2 PN rate, oocyte utilization rate, utilized embryos rate and cycle cancelation rate in the two groups were similar. However, the high-quality embryo rate was significantly higher in the ICSI group than those in the IVF group (p < 0.05), which is shown in Table 2. Finally, a total of 239 patients received embryo transfer. In ICSI and IVF group, the miscarriage rate and implantation rate were similar in the two groups. However, the clinical pregnancy rates were significantly higher in the ICSI group (55.56% vs. 40.98%, P < 0.05), as shown in Table 3.

**Table 2:** Embryonic outcome between the two groups.

	ICSI	IVF	ICSI vs. IVF
	n = 158 (47%)	n = 178 (53%)	p-Value
Oocytes per group (n)	11.28 ± 5.61	10.16 ± 4.83	0.051
MII per group (n)	8.24 ± 5.07	8.72 ± 4.51	0.355
2PN/oocytes (%)	69.12 (900/1302)	71.73 (1114/1553)	0.138
Utilized embryos per group (n)	2.18 ± 2.05	2.22 ± 2.17	0.837
High-quality embryos per group (n)	0.32 ± 0.67	0.16 ± 0.62	0.001*
Utilized embryos/oocytes (%)	26.42 (344/1302)	25.50 (396/1553)	0.578
Utilized embryos/2 PN (%)	38.22 (344/900)	35.55 (396/1114)	0.227
High-quality embryos/2 PN (%)	5.56 (50/900)	2.60 (29/1114)	0.001*
Cancel cycles/ART cycle (%)	25.32 (40/158)	26.40 (47/178)	0.901

Two-sample independent *t*-test or Person χ2 test.

*Significant difference between IVF and ICSI groups, P < 0.05.

**Table 3:** Clinical outcome between IVF and ICSI.

	ICSI	IVF	ICSI vs. IVF
	n = 117 (48.95%)	n = 122 (51.05%)	p-Value
Endometrial thickness (cm)	0.98 ± 0.16	0.94 ± 0.14	0.053
Number of embryos transferred	1.53 ± 0.73	1.49 ± 0.77	0.686
Clinical pregnancy rate (%)	55.56 (65/117)	40.98 (50/122)	0.028*
Implantation rate (%)	37.86 (78/206)	31.48 (68/216)	0.184
Miscarriage rate (%)	9.23 (6/65)	10.00 (5/50)	1.000

Two-sample independent *t*-test or Person χ2 test.

## 4 Discussion

The present study evaluated the impact of ICSI versus traditional IVF insemination on embryo quality in non-male infertile patients with poor-quality embryos in the previous IVF cycle. The results of this present study indicate that ICSI did not increase 2 PN rate, oocyte utilization rate, miscarriage rate and cycle cancelation rate of the patients with poor-quality embryos in the previous IVF cycle, however, it improved the high-quality embryo rate and clinical pregnancy rate. A previous study concluded that the more immature oocytes undergo a complete maturation during IVF, the higher the likelihood to fertilize spermatozoa in an incubator overnight [13]. However, in this present study, the absolute MII oocytes and 2 PN didn't differ significantly. There are two possible explanations for this phenomenon. Firstly, the experience of the doctor who monitored the COH protocol plays a critical role. Secondly, in IVF group 2 PB and MII oocyte were observed at 4–6 h after insemination. Therefore, oocytes matured in vitro were excluded. ICSI related to potential mechanical damage to the oocyte membrane and cytoplasm during the procedure, which can reduce the number of mature oocytes for fertilization. In addition, ICSI can result in lower fertilization. However, ICSI could increase the normal fertilization rates with one sperm injection and lower the multiple pronuclear rates in young or advanced age women [3]. The two reasons above may cause a similar fertilization rate in two groups. The results of the present study were consistent with the previous meta-analysis of the effect of ICSI in advanced age infertile couples with non-male factor [14]. In present study, the insemination technique did not affect the normal fertilization rate and oocyte utilization rate. However, the ICSI group had a significantly higher high-quality embryos rate compared to the IVF group. Similarly, Peng N. et al. studied ICSI in the advanced age patient to increase the number of high-quality embryos [8]. Yang D et al. also found a similar fertilization rate in the IVF and ICSI groups, however, embryo quality and clinical outcomes were higher after ICSI [15]. ICSI was superior probably because it can avoid oocyte and zygote cultured with a lot of spermatozoa and reduced the exposure to the reactive oxygen species, which were produced by spermatozoa. Oxidative stress impairs sperm motility and causes single-strand DNA break of sperm, contributing to the clinical outcome [16]. Moreover, appropriate ICSI techniques can trigger artificial calcium (Ca2+), which plays a critical role in embryo cleavage and development [17,18]. Whereas, some studies demonstrated the ratio of high-quality embryos/total embryos were similar and high-quality blastocyst rate was higher in the IVF group than the ICSI group [19]. Some other previous studies were consistent with the present study [4,20]. However, one of the main causes is that only patients with poor-quality embryo in the previous IVF cycle were included in the present study, implying a higher risk of selection bias. The risk of selection bias on subjects and insemination method influenced the corresponding reproductive outcomes. Furthermore, the skill of the embryologist could be another factor affecting the success rates of ICSI. The study by Blake et al. revealed that the location of the injection affected the embryo quality, with injection near the meiotic spindle causing a lower embryo quality on day 3 [21]. Moreover, sperm morphology has implication for laboratory and clinical outcomes [22]. Therefore, in our center, ICSI was performed by experienced embryologists. It is notably that the ICSI group can't increase oocyte utilization rate in this present study, possibly contributed by the intrinsic factor of gametes. The patients with poor-quality embryo in the previous IVF cycle, are more likely to have a poor prognosis in following cycles. In the present study, good egg retrieval and normal fertilization was achieved in this subpopulation of patients, however, utilized embryos was limited. A previous study showed that embryo quality depends on the intrinsic factor of the gametes, such as oocyte cytoplasmic factors, maternal-effect mutations and sperm DNA fragments, rather than the fertilization methods [23].

Regarding the clinical outcome of the two groups, the significant higher clinical pregnancy rate was found in ICSI group. Embryo quality is a major factor influencing the probability of pregnancy in the context of ART. The present study investigated the laboratory treatment strategies for the specific group of patients with poor-quality embryo in the previous IVF cycle, and the results suggested that ICSI improved the embryo quality, selecting ICSI maybe helpful for this type of patients.

The main limit of study was the retrospective design. There are many confounding biases, so it would have been credible if it had used sibling data. A further prospective clinical trials study on delivery rate may provide more conclusive answers.

In conclusion, ICSI may be superior to IVF for the subpopulation with poor-quality embryo in the previous IVF cycle. Although ICSI didn't increase the 2 PN rates, oocyte utilization rate, miscarriage rate and cycle cancelation rate, it increased high-quality embryo rates and clinical pregnancy for such subpopulation. Prospective randomized controlled trials are further needed to verify this opinion.

## Funding

This work was supported by: The Women and Children's Hospital of Chongqing Medical University (grant number: 2021YJQN07) and Chongqing Natural Science Foundation of China (grant number: CSTB2022NSCQ-MSX0253).

## Ethics approval and consent to participate

This study was approved by the Ethics Committee of Women and Children's Hospital of Chongqing Medical University (number: 2020-RGI-09).

## Author contribution statement

Jiang Wang: conceived and designed the experiments and wrote the paper.

Shun Xiong; Yang Gao: analysed and interpreted the data.

Fei Xia; Biao Wei; Jiayi Zou: performed the experiments.

Guoning Huang; Wei Han: contributed reagents, materials, analysis tools or data.

## Data availability statement

Data will be made available on request.

## Declaration of competing interest

The authors declare that they have no known competing financial interests or personal relationships that could have appeared to influence the work reported in this paper.
